# PSMA Theranostics: Science and Practice

**DOI:** 10.3390/cancers13153904

**Published:** 2021-08-02

**Authors:** Kgomotso Mokoala, Ismaheel Lawal, Thabo Lengana, Mankgopo Kgatle, Frederik L. Giesel, Mariza Vorster, Mike Sathekge

**Affiliations:** 1 Department of Nuclear Medicine, University of Pretoria, Pretoria 0001, South Africa; kgomotso.mokoala@up.ac.za (K.M.); u14446792@tuks.co.za (I.L.); mariza.vorster@up.ac.za (M.V.); 2 Nuclear Medicine Research Infrastructure (NuMeRI), Steve Biko Academic Hospital, Pretoria 0001, South Africa; mankgopo.kgatle@up.ac.za; 3 KVNR Molecular Imaging, Pretoria 0001, South Africa; u14446325@tuks.co.za; 4 Department of Nuclear Medicine, University Hospital Duesseldorf, 40210 Duesseldorf, Germany; Frederik.Giesel@med.uni-duesseldorf.de

**Keywords:** prostate cancer, theranostics, PSMA, ^177^Lu, ^225^Ac

## Abstract

**Simple Summary:**

A significant number of prostate cancer patients will progress to metastatic castrate resistant prostate cancer despite optimal therapies. There is a growing need for alternative therapeutic strategies for this category of patients. Theragnostic refers to the ability to use an organ specific ligand and label it to both a diagnostic/imaging and therapeutic agent. Several prostate specific membrane antigen radioligands have been developed for imaging and treating PCa. Beta and alpha emitting radionuclides have been used with great success. Xerostomia is the greatest adverse event associated with radioligand therapy. More trials are necessary to determine the timing of introducing these novel therapies and to assess the efficacy as monotherapy as well as in combination with other novel agents to improve efficacy and reduce side effects to other organs.

**Abstract:**

Prostate cancer (PCa) causes significant morbidity and mortality in men globally. While localized PCa may be managed with curative intent by surgery and/or radiation therapy, the management of advanced hormone resistant metastatic disease (mCRPC) is more challenging. Theranostics is a principle based on the ability to use an organ specific ligand and label it to both a diagnostic and a therapeutic agent. The overexpression of prostate specific membrane antigen (PSMA) on prostate cancer cells creates a unique opportunity for development of targeted radionuclide therapy. The use of both beta and alpha emitting particles has shown great success. Several clinical trials have been initiated assessing the efficacy and safety profile of these radionuclide agents. The results are encouraging with PSMA directed radioligand therapy performing well in patients who have exhausted all other standard treatment options. Future studies need to assess the timing of introduction of these radionuclide therapies in the management schema of mCRPC. Drugs or therapies are not without side effects and targeted radionuclide therapies presents a new set of toxicities including xerostomia and myelosuppression. New therapeutic strategies are being explored to improve outcomes while keeping toxicities to a minimum. This review aims to look at the various PSMA labelled tracers that form part of the theragnostic approach and subsequently delve into the progress made in the area of radionuclide therapy.

## 1. Introduction

Prostate cancer (PCa) is the second most frequent cause of cancer-related mortality in men globally. Therapy of PCa is a collaborative effort based on a multidisciplinary tumour board approach together with the patient and it is guided by the available resources. The widespread availability of screening tests has improved the early diagnosis of organ-confined PCa, making definitive therapy feasible [1]. Low risk organ confined disease may be managed in one of three ways, namely watchful waiting and active surveillance, surgery and/or radiation therapy (external beam therapy or brachytherapy). In localized high-risk or locally advanced disease, combination therapy with radical radiation therapy and long-term androgen deprivation therapy (ADT) with or without docetaxel is advised. Hormone sensitive metastatic disease is best managed with the combination of ADT and second generation anti-androgens (abiraterone acetate, enzalutamide, apalutamide and darolutamide) or ADT and docetaxel [2,3]. At some point during the course of the disease, PCa becomes resistant to ADT and is considered Metastatic Castrate-Resistant Prostate Cancer (mCRPC). Five years from the time of diagnosis, 10–20% patients develop mCRPC [4]. First line therapy for castrate resistant disease is second generation anti-androgens, docetaxel and ^223^Radium chloride (for patients with bone metastases only). Failure to respond to docetaxel may be treated with the second line agent, Cabazitaxel [3]. Metastatic cancer is one of the leading causes of prostate cancer-associated deaths. Despite great initial responses, patients soon progress and have limited treatment options.

**Figure 1:**
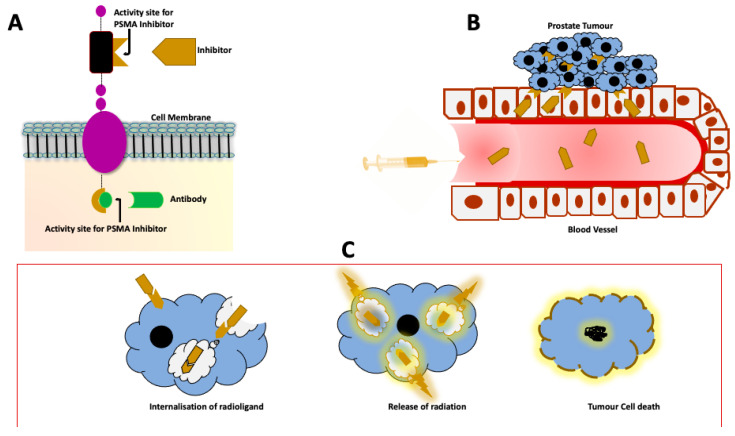
Illustration of theragnostic approach to the management of PCa. (**A**) The PSMA protein with the different components, namely the intracellular, transmembrane and the extracellular portion. The activity sites on both the intracellular and extracellular domains have been used for binding of monoclonal antibodies, minibodies and small molecules. This enables targeting of the PSMA protein for both imaging and therapy. (**B**) Once the tracer is injected into the bloodstream, the PSMA ligand binds to the activity site on the tumour cells. (**C**) After binding to the activity site on the cell membrane, the radioligand is internalized and releases radiation from within the cell. The end product is DNA damage with resultant tumour cell death.

Theranostics or theragnostics resulted from the amalgamation of the words therapy or therapeutic and diagnostics. This term was coined by Funkhouser in 2002 and has been the catchword since 2011 [5]. In the true sense this term refers to a close relationship between diagnostics and therapy whereby an appropriate diagnostic methodology is utilized to individualize therapeutic interventions [6]. Other definitions have expanded it to include monitoring of treatment response. In the discussion of the role of Nuclear Medicine in the management of prostate cancer, theranostics refers to the use of molecules that target disease-specific structures in the tumour and metastases. These molecules can be combined with an imaging agent for diagnosis/staging and subsequently to a therapeutic agent for therapy of the identified disease. Figure 1 below is an illustration of the practical aspects of theragnostics.

## 2. Prostate Specific Membrane Antigen (PSMA)

The emergence of Prostate Specific Membrane Antigen (PSMA) as a theragnostic agent has completely revolutionized the management of PCa. The PSMA protein is also known as glutamate carboxy peptidase II and folate hydrolase 1. It is a transmembrane glycoprotein enzyme found on the cell surface. The PSMA protein is divided into three parts, namely the internal portion, transmembrane portion, and the external portion [7,8]. The extracellular portion makes up 95% of the PSMA protein and is the accessible target for both small molecules and antibody-based agents for imaging and therapy. The internalization process that allows endocytosis of bound proteins on the cell surface of the PSMA receptor allows PSMA labelled radioisotopes to be concentrated within the cell [9]. The term “prostate specific” is a misnomer as there is minimal expression of PSMA in normal prostate tissue, kidneys, duodenum, salivary and lacrimal glands, brain, and intestines; however, it has been found to be highly expressed (up to 1000-fold) in PCa [10,11,12,13,14]. There is a strong correlation between PSMA expression and both the Gleason score and serum PSA value [15]. PSMA expression is decreased significantly during ADT confirming that PSMA expression is regulated by Androgen receptors [16]. There has been an evolution of the PSMA based approaches from antibodies and nanobodies to small molecules. The studies that provided the proof of concept for the utilization of PSMA targeting agents employed a mouse monoclonal antibody against the intracellular domain of PSMA protein. This was labelled to Indium-111 (^111^In-capromab pendetide) for Single Photon Emission Tomography (SPECT) imaging and marketed as Prostascint^®^. While it had great correlation to tissue specimen, it suffered from a lack of sensitivity [17,18,19,20]. This tracer was approved by the FDA, however it never had great take-off because of the slow kinetics which resulted in prolonged imaging duration and poor-quality images. Another antibody targeting the extracellular component (J591) was discovered by Dr Bander and colleagues. Other anti-PSMA monoclonal antibodies that bind various epitopes of the PSMA protein have also been investigated and include J415, Hybritech PEQ226.5 and PMJ2J004.5. They have been labelled with ^111^In, ^124^I, ^64^Cu and ^89^Zr and have demonstrated great promise with improved targeting of nodal, soft tissue and skeletal metastases [21,22,23]. Despite these modifications, there has been limited practical use of these tracers because they are still antibodies and suffer the same weaknesses as the ^111^In labelled antibody. Over the years PSMA-targeted small molecule ligands have been developed and have made significant progress. They have been labelled to ^99m^Tc, ^68^Ga and ^18^F for imaging as well as ^177^Lu, ^222^Bi and ^225^Ac for therapy.

## 3. PSMA Based Imaging

Since the first human experience with PSMA small molecules in 2008 with the use of Iodine-123, there has been great progress made in this space with multiple variants of small molecule PSMA inhibitors being utilized for PCa imaging. The emergence of these tracers has resulted in better sensitivity and specificity for detecting locoregional and distant metastasis [24]. Although the bulk of data published is on Positron Emission Tomography (PET) imaging with PSMA ligands, there are a few SPECT tracers that have been investigated.

## 4. SPECT Tracers

The first SPECT tracers utilizing small molecules to image PCa were ^123^I-(MIP)-1072 and ^123^I-(MIP)-1095. Both these agents could detect the local tumour and metastases in soft tissue and bone with excellent tumour to target contrast [25,26]. The first human therapy utilizing PSMA ligands resulted from the use of these ligands substituting the ^123^I with ^131^I [26]. Technetium (^99m^Tc) is a very convenient isotope because it is widely available and inexpensive. It has a 6-h half-life and can be labelled to a variety of tracers. There are a host of ^99m^Tc labelled PSMA ligands (^99m^Tc-MIP-1404, ^99m^Tc I & S PSMA, ^99m^Tc-HYNIC-PSMA) that have been evaluated for imaging of PCa [27,28,29,30,31]. All published work demonstrates that these tracers are useful in imaging of PCa. Caution should be applied especially in settings of low serum PSA and low volume disease; however, the value of these tracers should not be underestimated. While the tracers used for PET/CT have been shown to have greater imaging properties and results, these facilities may not always be affordable or available to remote areas. The advantage of SPECT tracers, more so ^99m^Tc labelled PSMA ligands is that they may be of value in radioguided surgery with intraoperative probe localization [32,33,34].

## 5. PET Tracers

PET imaging of PCa with either Gallium-68 or Flourine-18 tracers is the most widely studied and validated method. A few trials have been undertaken to assess the diagnostic ability of PET labelled PSMA radiopharmaceuticals for PCa. Gallium-68 labelled tracers are the most extensively investigated.

### 5.1. Gallium-68 Labelled PSMA Ligands

Gallium-68 (^68^Ga) is a PET tracer with favourable pharmacokinetic characteristics that enable labelling with many peptides and other small molecules. The commercial accessibility of germanium-68/gallium-68 (^68^Ge/^68^Ga) generators and more recently cyclotron produced ^68^Ga, has resulted in vast data and publications focused on PSMA PET ligands. The ^68^Ga labelled tracers have been the most validated and as such the European Association of Urology and National Cancer Comprehensive Network have included ^68^Ga-PSMA PET/CT in their guidelines [1,35].

A prospective multicenter study addressed the use of ^68^Ga-PSMA-11 PET/CT in patients with newly diagnosed PCa and negative bone scan findings. The use of PET/CT resulted in a change in management in 12.6% of patients with a sensitivity and specificity of 41.5% and 90.9% respectively [36].

The multicenter prospective 2 phase proPSMA study set to compare conventional imaging by CT and bone scan against PSMA PET/CT in men with high risk PCa. A greater diagnostic accuracy was noted with PSMA PET/CT than conventional imaging (92% (range of 88–95%) vs. 65% (range of 60–69%); *p* < 0001) [37,38]. The sensitivity and specificity of PSMA PET/CT were also higher [37,38]. The imaging findings led to a change in management with a higher frequency in the PSMA PET/CT group than the conventional imaging cohort. This study provides convincing evidence and further confirms the previous findings of other authors [39]. These studies are helping cement the value of PSMA PET/CT prior to definitive therapy seeing as it impacts on the management of PCa.

A recent systematic review and meta-analysis to assess the diagnostic utility, sensitivity, and specificity of ^68^Ga-PSMA PET in advanced prostate cancer found an improved detection in patients with biochemical recurrence. In this analysis with close to five thousand patients from 37 articles/studies, in the setting of biochemical recurrence, there was a significant in difference in scan positivity in the prostate bed of patients who had radical prostatectomy (22%) or radiotherapy (52%). The detection at low PSA values < 0.2 ng/mL was 33% while for PSA values between 0.2 and 0.5 ng/mL were 45% [24].

There is mounting evidence to support the use of PSMA based imaging in the staging of high-risk patients as well as in patients with biochemical recurrence. These ^68^Ga labelled tracers have been shown to outperform conventional imaging and should form an integral part of diagnostic algorithms or guidelines.

### 5.2. Fluorine Labelled PSMA Ligands

Flourine-18 (^18^F) is a cyclotron produced radionuclide with favourable nuclear and physical properties. It has a high positron decay ratio (97%), relatively short half-life (109.7 min), and a low positron energy (0.635 MeV) which results in increased resolution because of the short diffusion range [40]. The half-life makes ^18^F more amenable for commercial development because it can be produced centrally and distributed to satellite sites. ^18^F based agents developed for PCa imaging include ^18^F-DCFBC, ^18^F-DCFPyl, ^18^F-rhPSMA and ^18^F-PSMA-1007. The Food and Drug Association (FDA) recently approved the use of ^18^F-DCFPyl in the work-up of PCa patients.

In their prospective study of 130 men with biochemical recurrence, Rousseau et al. localized recurrent disease in 60% of cases with PSA between ≥0.4 and <0.5, 78% with PSA between ≥0.5 and <1, 72% with PSA between ≥1 and <2 and 92% with PSA ≥ 2. They reported that there was a change in treatment intent, change in the stage of the disease and change in management plans in 65.5%, 65.5% and 87.3% of patients, respectively [41].

In a study by the National Cancer Institute (NCI), the sensitivity of ^18^F-DCFPyl in 90 patients with biochemical recurrence was 47.6% in patients with PSA level between 0.2 and 0.5 ng/mL, 50% in patients with PSA 0.5–1 ng/mL, 88.9% for PSA between 1–2 ng/mL and 94% in patients with PSA > 2 ng/mL. They validated their findings against histology or a composite reference of histology, imaging (pelvic magnetic resonance imaging (MRI) performed within 3 months of the PSMA PET), follow-up CT, ^99m^Tc bone scan or ^18^F-sodium fluoride PET/CT at 3 to 6 months) or clinical follow-up (PSA trend after treatment). When using histopathology for validation, the positive predictive value (PPV) on a per patient basis was 93.3% while it was 96.2% when using the combination of histology, imaging and/or clinical follow-up [42]. Although ^18^F-DCFPyl has a high positivity rate even at low PSA values, it performs better as the PSA value rises. This is comparable to published results using ^68^Ga-PSMA ligands.

The fact the ^18^F-PSMA-1007 is predominantly excreted by the hepatobiliary system theoretically implies amended biodistribution which will lead to improved detection in the pelvis or prostate bed region as compared to the other PSMA PET agents. A systematic review by Awenat and colleagues found a high diagnostic accuracy of ^18^F-PSMA-1007 of 80–100% on a per patient-based analysis and from 93–95% on a per lesion-based analysis in initial staging of PCa patients [43]. When compared with other radiological and scintigraphic imaging methods ^18^F-PSMA-1007 had a higher sensitivity, whereas when compared to other PSMA targeted PET/CT tracers, it had a similar diagnostic accuracy for initial staging. In the setting of biochemical recurrence, where it is purported that ^18^F-PSMA-1007 will have an added advantage, a multi-centre study found high detection rates which were comparable to or better than ^68^Ga-PSMA ligands. The detection rates had a positive correlation with the serum PSA level. Local recurrence was revealed in 24.7% of the patients, while 40.6% of the patients had pelvic lymph node metastasis and 43.8% of patients had bone and visceral metastasis [44].

A systematic review and meta-analysis of six ^18^F-PSMA PET/CT articles/studies with 645 patients with biochemical recurrence, found a pooled detection rate of 81% (95% CI: 71–88%). Using different PSA cutoff values, the pooled detection rate was 49% for PSA < 0.5 ng/mL (95% CI: 23–74%) and 86% for PSA ≥ 0.5 ng/mL (95% CI: 78–93%). The detection rate is highly dependent on PSA values with an overall better detection rate with higher PSA values ≥ 0.5 ng/mL [45].

The choice of tracer should be guided by local circumstances including health funding models/coverage, availability of skilled personnel for onsite radiolabelling for ^68^Ga or availability of local producers of ^18^F-PSMA tracers and, of course evidence and personal experience with the different tracers.

### 5.3. Indications for PSMA Ligand Imaging

On 1 December 2020, the FDA approved ^68^Ga-PSMA-11 for imaging in two clinical settings, namely in men with suspected metastases who are candidates for initial definitive therapy and secondly in men with suspected recurrence based on elevated serum PSA levels [46].

On the other hand, the European Society of Medical Oncology (ESMO) has recommended the use of PSMA PET/CT imaging in biochemical recurrence because of the superior sensitivity and specificity compared to conventional imaging [3]. While PSMA PET/CT has better sensitivity and specificity than CT or bone scan, the ESMO does not make a specific recommendation for its use in patients with intermediate or high-risk disease as it has not been shown to improve clinical outcomes [3].

The joint European Association of Nuclear Medicine (EANM) and Society of Nuclear Medicine and Molecular Imaging (SNMMI) guideline for PSMA ligand imaging is based on data from studies using ^68^Ga-PSMA. Currently the indications for imaging with ^68^Ga-PSMA or other PSMA labelled radioligands is as follows [47]:

- To localize tumour in biochemical recurrence, especially with PSA values between 0.2 and 1 ng/mL,
- High risk (Gleason score > 7, PSA > 20 ng/mL and clinical stage T2c-3a) patients to guide clinical decision making and for therapy planning,
- Staging before and during PSMA-directed therapy in patients with mCRPC,
- Targeted biopsy in patients with high suspicion of PCa with previous negative biopsy,
- Monitoring systemic therapy in metastatic Pca.

Of these indications, the first two have been validated while the last three are emerging applications. With the widespread introduction of radionuclide radiotherapy, the use of imaging for monitoring therapy will become routine.

The growth and expansion of the role of PSMA based ligands in imaging and therapy has also called for imaging specialists to adapt and formulate methods or algorithms for reporting follow-up imaging when monitoring patients that have had local or systemic therapies. This is for standardization and harmonization. In patients with mCRPC, it has been proposed that molecular response assessment may be better than morphological response criteria, especially in bone-dominant disease. In a consensus statement by international experts in PCa, the panelists agreed that patients should be broadly categorized as responders or non-responders. Patients with complete response (CR), partial response (PR), or stable disease (SD) on PSMA PET/CT should be classified as responders, while those with progressive disease (PD) on PSMA PET/CT should be considered as non-responders. The criteria to classify PSMA response into one of the four categories (CR, PR, SD, PD) mentioned above include [48,49]:

- Complete response: Disappearance of any lesion with tracer uptake
- Partial response: Reduction of uptake tumour PET volume by >30%
- Stable disease: Change of uptake and tumour PET volume by ±≤30% and no new lesions documented
- Progressive disease: Appearance of ≥2 new lesions or increase of uptake or tumour volume ≥30%

In the category of progressive disease, the panelists made special exceptions in the scenario of oligo- or polymetastatic disease. When performing follow-up imaging in patients that have had PSMA radioligand therapy, caution should be applied. This is because during the course of disease, there may be downgrading of tumour cell PSMA expression. Response assessment should be based on a composite of clinical, biochemical, and other imaging findings.

### 5.4. ^18^F-FDG for Imaging Discordance

Well differentiated early-stage prostate cancer cells demonstrate low tracer (^18^F-FDG) avidity. Prostate cancer is heterogenous in nature and in advanced mCRPC when there is de-differentiation, this heterogeneity is even more pronounced. There is reduced expression of PSMA molecule in advanced disease with reduced to no uptake/avidity on ^68^Ga-PSMA ligand imaging. These patients have been found to have discordance between ^68^Ga-PSMA and ^18^F-FDG PET/CT imaging and more aggressive phenotype with poor response to PSMA directed therapies [50,51,52,53]. Chen et al. found 13/56 (23.2%) of patients with at least one PSMA negative and FDG positive lesion. The PSA and Gleason score were higher in these patients [54,55]. Therefore, it may be of value to add ^18^F-FDG in the imaging algorithm of patients with mCRPC who have negative PSMA imaging despite rising serum PSA level. In the Australian TheraP trial, dual imaging with ^68^Ga-PSMA and ^18^F-FDG was performed. This was to guide the selection of patients for radiolabelled radionuclide therapy as patients with PSMA negative and FDG positive studies were excluded [56,57].

## 6. PSMA Radioligand Therapy

Since the discovery of PSMA theranostics with Iodinated PSMA molecules, there has been a plethora of publications on the subject of PSMA based therapy for PCa. Over the years modifications to the molecules have resulted in better tumour uptake and responses and several trials have now released the results of such therapies. These include anti-PSMA antibodies such as J591 and PSMA targeted small molecules ligands such as MIP-1095, PSMA I & T, and PSMA-617 labelled with alpha or beta particle emitting isotopes. The optimal setting to use/apply these therapies is still being sought, hence the multiple clinical trials. Currently, these therapies are usually indicated in patients with metastatic, castrate resistant PCa (mCRPC) who have exhausted all other treatment options or are ineligible for those treatment options. The condition for therapy with PSMA ligands is that there should be adequate uptake on the PSMA ligand based diagnostic scan. Adequacy of uptake is not standardized however the study by Hofman and colleagues suggested uptake in lesions that is 1.5 times higher than liver uptake [58,59].

### Practical Aspects of Radionuclide Therapy with ^177^Lu-PSMA

The decision to proceed with radionuclide therapy incorporates imaging findings (PSMA based ligand imaging) as well as clinical and biochemical findings. Ideally, this decision is made by a multidisciplinary tumour board.

**Figure 2:**
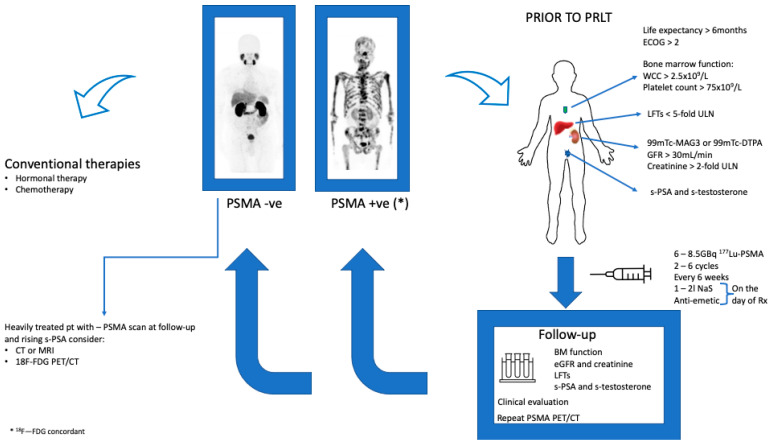
Algorithm of the practical aspects of therapy from the selection of patients for targeted radionuclide therapy to the therapeutic procedure and follow-up. * ^18^F—FDG Concordant disease.

The European Association of Nuclear Medicine have published a guideline for PSMA radioligand therapy in patients with mCRPC. These guidelines provide a base for the harmonization of molecular based therapy with ^177^Lu-PSMA. Figure 2. Below is an algorithm for the therapeutic procedure as well as the follow-up of patients treated with ^177^Lu-PSMA.

## 7. Lutetium Therapy

The most popular radionuclide for therapeutic applications across a wide spectrum of malignancies is ^177^Lu. The successful radiolabelling of ^177^Lu to a somatostatin analogue for the treatment of neuroendocrine tumours has led to the worldwide growth in interest and use of this radionuclide in therapy [60]. It is a radiometal that is reactor produced and a beta emitter (β-emitter) with a maximum energy of 498 keV (±0.5 MeV). It also has gamma rays (y-rays) with energies of 208 and 113 keV (11% and 6%, respectively) which allow for imaging and dosimetric calculations [61]. The half-life of ^177^Lu is 6.73 days making it long enough to impart the radiation to the tumour over an extended period of time. It has a maximal and mean tissue penetration of <2 mm (1.7 mm) and 0.23 mm, respectively which makes it ideal for small tumours. The tissue penetration of ^177^Lu is equivalent to 20 and 60 cell diameters, which results in a “crossfire effect”, that is DNA damage in the tumour cells as well as the surrounding cells. Of the various PSMA small molecule peptides and antibodies that have been labelled with ^177^Lu, PSMA-DKFZ-617 is the most widely published with PSMA I & T also fast gaining publicity and to a lesser degree, the antibody J591 also being investigated [61,62,63,64,65,66,67,68,69,70].

### 7.1. Efficacy of ^177^Lu-PSMA in mCRPC

One of the first studies to provide initial evidence of the safety and efficacy of PSMA labelled radioligand therapy is from wo-center study in Germany. In ten patients with mCRPC, seven (70%) experience a PSA decline 2 months after therapy with five (50%) having a PSA decline >50% after a single dose of ^177^Lu-DKFZ-617 [71]. The side effect profile included mild haematological toxicity, insignificant acute renal toxicity and transient xerostomia. The results of this early work are encouraging and offered some insight to the potential use of ^177^Lu-PSMA in the treatment of mCRPC.

The efficacy of ^177^Lu-PSMA-617 was further highlighted in the early work by Rahbar and colleagues who assessed the response and toxicity after a single dose of ±6 GBq ^177^Lu-PSMA-617. In this retrospective multicenter analysis, the authors found that there was a PSA decline in 35/74 patients (64%) with 31% of patients having a PSA decline of greater than 50%. These results were not confirmed by a second measurement; however, the results still demonstrated the potential efficacy of ^177^Lu-PSMA-617 as a therapeutic option for patients with mCRPC. Unfortunately, seventeen patients (23%) had progressive disease as evidenced by a PSA level rise of more than 25%. The patients in the study had extensive metastatic disease and had been heavily pre-treated [72].

Much of the initial work on ^177^Lu-PSMA radionuclide therapy is based on retrospective analysis of data acquired in centers that were offering ^177^Lu on a compassionate basis. A German multicenter study investigating ^177^Lu PSMA-617 therapy in pooled data of 145 men with mCRPCa demonstrated that this therapy is effective and safe. All patients had been pre-treated with standard of care regimen including 1st and 2nd line chemotherapy. With administered doses ranging between 2–8 GBq, there was a biochemical response (>50% decline in PSA) in 40% of the patients after the first cycle with response in 45% of patients over the entire follow-up period [73]. The authors concluded that this therapy may supersede the performance of other third-line systemic therapies. Interestingly, a metanalysis of 16 studies with a total of 671 patients also found a biochemical response in 45% of the patients and any decline in PSA in 75% of patients [74]. They went on to report on the progression free survival (PFS) and overall survival (OS) and found that the median PFS was 11 months (IQR: 7.6–13.7) from the five studies that reported PFS, while the median OS from six studies was 13.7 months (IQR: 8–14) [74]. Another meta-analysis also found a similar pooled PSA response (>50% decline) in 34.4% (95% CI: 30.14–38.97%) of patients [75].

In their single arm, single center prospective study, Hofman et al. proved that ^177^Lu-PSMA-617 is effective with high response rates and minimal side effects in patients with mCRPC who had progressed after conventional therapies. Thirty men fulfilled the criteria for inclusion into this study which included patients with progressive disease with high PSMA expression as evidenced by positive PSMA PET and negative FDG PET. Study participants received up to 4 cycles of ^177^Lu-PSMA-617 at six weekly intervals. The primary end point was PSA response and toxicity with imaging response and quality of life response being additional primary end points. All patients had been heavily treated with atleast one line of either chemotherapy or androgen axis targeting drugs. With a mean administered activity of 7.5 GBq per cycle, just over half (17/30) of the patients achieved a PSA decline (57%; 95% CI: 37–75). The median PSA PFS was 7.6 months (95% CI: 6.3–9.0) and OS was 13.5 months (95% CI: 10.4–22.7). While there was treatment related toxic effects to the salivary gland and bone marrow, none of the patients dies because of the therapy. The commonest side effect was grade 1 xerostomia (87%) with 4/30 (13%) patients presenting with grade 3 or 4 thrombocytopaenia as a result of the therapy [58].

Another trial to emerge from the same Australian group compared ^177^Lu-PSMA-617 to cabazitaxel in terms of efficacy and safety. It was a multicenter, phase II randomized, stratified, two-arm clinical trial in patients with mCRPC suitable for chemotherapy. Patients in the treatment arm receive standard of care treatment with cabazitaxel (20 mg/m^2^) intravenously every 3 weeks with Prednisolone 10 mg orally daily for a maximum of 10 cycles. The experimental group received 8.5 GBq ^177^Lu-PSMA-617 decreasing by 0.5 GBq with every subsequent cycle and this was repeated every 6 weeks up to a maximum of 6 cycles. The primary end point was to determine the proportion of patients with PSA response in both groups with OS and objective tumour response rate, PFS, PSA PFS, rPFS and pain response as some secondary end points. Sixty-five of 99 men (66%) randomized to ^177^Lu-PSMA-617 achieved PSA response compared with 31 of 85 men (37%) randomized to cabazitaxel. Treatment with ^177^Lu-PSMA-617 delayed progression as compared with cabazitaxel (Hazard ratio, HR = 0.63, 95% CI = 0.46–0.86, *p* = 0.0028). Among 78 men with evaluable lesions as per RECIST criteria, objective response occurred in 49% (95% CI = 33–65) of patients treated with ^177^Lu-PSMA-617 compared with 24% (95% CI = 11–38) of patients treated with cabazitaxel (Hofamn). This study also showed that ^177^Lu-PSMA therapy is safe as evidenced by 33% grade 3/4 adverse events as opposed to 53% in the cabazitaxel group [59].

### 7.2. Vision Trial Results

The results of the much-anticipated Phase III VISION trial are finally in and have been officially presented during the 2021 American Society of Clinical Oncology Annual Meeting Plenary session. The trial recruited 831 men with metastatic PCa and randomized them to two arms to receive either best standard of care (bSOC) therapy or best standard of care plus ^177^Lu-PSMA-617 targeted radionuclide therapy [76,77]. The conclusion is that bSOC plus ^177^Lu-PSMA-617 significantly improves overall survival than bSOC alone. The median OS for the ^177^Lu-PSMA-617 + bSOC arm was 15.3 months compared to 11.3 months in the bSOC only arm, with a 38% (hazard ratio: 0.62; 95% CI: 0.52–0.73) reduction in risk of death (median OS benefit of 4 months). Similarly, the median radiographic progression free survival (rPFS) was prolonged in the ^177^Lu-PSMA-617 arm than the bSOC with a median rPFS of 8.7 months (99.2% CI: 7.9–10.8) and 3.4 months (99.2% CI: 2.4–4), respectively. Consistently, the disease control rate and overall response rate were higher in the ^177^Lu-PSMA-617 and bSOC cohort than in the bSOC cohort. Unfortunately, the ^177^Lu-PSMA-617 cohort had higher rate of treatment related adverse events compared to the bSOC only cohort, 85.3% vs. 28.8%, respectively [76]. These results are likely to influence management decisions and future guidelines. The combination of the bSOC and ^177^Lu-PSMA-617 has demonstrated superior results but at the risk of increased treatment related side effects, therefore it may be worthwhile to consider a head-to-head comparison of ^177^Lu-PSMA-617 monotherapy against the conventional therapies or bSOC therapy [77]. The TheraP trial has already demonstrated superiority of ^177^Lu-PSMA against carbazitaxel, therefore the evidence is accumulating and should influence decision-makers to consider the introduction of ^177^Lu-PSMA in guidelines and protocols for the management of mCRPC.

### 7.3. Indicators of Prognosis

Several factors have been shown to be predictive of treatment response and overall survival. Based on imaging parameters on pre-therapeutic PSMA PET imaging, Seifert et al. found that the PSMA tumour volume (PSMA-TV) and PSMA tumour lesion quotient (PSMA-TLQ) were independent prognostic factors of survival [78]. Another study aimed to retrospectively identify pre-therapeutic imaging parameters that would predict which patients will achieve an early biochemical response to ^177^Lu-PSMA-617 therapy. Interestingly, this study found pre-therapeutic SUVmax (HR = 7.94; *p* = 0.001) to be correlated with a PSA change after two cycles while the PSMA-TV and whole-body total lesion (TL) PSMA were not [79]. The change in serum PSA has been shown to be the single most significant parameter correlated with overall survival. Patients with a PSA decline 2 months after the first cycle had a significantly longer median overall duration of survival (median: 63–71 weeks) than those that did not demonstrate any change in PSA (median: 33–47 weeks) [72,80,81]. The reports of the association between baseline PSA (prior to therapy) and overall survival have yielded conflicting results with other authors sighting a significant correlation between baseline PSA and overall survival following ^177^Lu-PSMA-PRLT [82,83]. Too much heterogeneity and controversy exist over the correlation between cumulative dose or administered activity with overall survival. Rahbar and colleagues indicated that a cumulative injected activity of ≥18.8 GBq is prognosticator of overall survival. Other independent predictors of biochemical response include an alkaline phosphatase (ALP) level < 220 U/L, the absence of visceral metastases and a higher number of therapy cycles [72]. The effect of prior therapies on overall survival was also shown by the results from the WARMTH multicenter study [84]. The presence of liver metastasis has been consistently reported to be associated with a poorer prognosis [85,86,87]. While the data is robust for the association between OS and the presence of liver metastasis, there is insufficient data on lung and brain metastasis, however a systematic review by von Eyben et al. found that patients with bone and lung metastases lived longer than patients with liver metastases [88].

### 7.4. Dosimetry

Dosimetric calculations require sequential imaging over several time-points, preferably using SPECT/CT techniques. Scan should be performed after 4–7 days post therapy. In patients with multiple sites of disease, estimation of clinically relevant tumour-absorbed dose is challenging [89]. Tolerance limits for the target organs, namely salivary glands, kidneys, and red marrow are 35 Gy, 28–40 Gy, and 2 Gy, respectively [89]. A dosimetric study of 26 participants imaged over a period of 7 days demonstrated that the 24-h post-injection scan had the highest target to background ratio for all normal organs whereas the 48-h image had the highest tumour to background ratio. The mean absorbed dose to the salivary gland, kidneys and red marrow were 1.24 mGy/MBq, 0.99 ± 0.31 mGy/MBq, and 0.04 mGy/MBq, respectively [90]. A higher absorbed dose was seen in patients with extensive skeletal metastasis, which is expected considering the path length of the beta particles of ^177^Lu. The infusion of amino acids for renoprotection does not seem to be beneficial as some groups omitted them and still found similar estimates for absorbed radiation dose to the kidney (0.88 ± 0.40 mGy/MBq and 0.6 Gy/GBq) [74,91,92,93]. Patients receiving less than 10 Gy were unlikely to achieve a fall in PSA of at least 50% [94]. In a prospective phase II trial designed to determine the dose that will be both effective and low on toxicity, Calais et al. randomized patients with mCRPC to receive either 6 GBq or 7.4 GBq of ^177^Lu-PSMA-617. The efficacy profile ^177^Lu-PSMA appeared to be favourable and comparable at both activity regimen. The PSA PFS was 2.9 months (95% CI: 0–9) in the 6 GBq group and 3.7 months (95% CI: 1.9–5.6) in the 7.4 GBq group. At the end of follow-up there were almost equal numbers of patients that had died in both treatment dose arms (86% vs. 87% for 6 GBq and 7.4 GBq, respectively) [95].

### 7.5. Toxicity

Systemic therapies are more likely to result in toxicities and ^177^Lu-PSMA is no exception. Early reports of radioligand therapy with ^177^Lu-PSMA have demonstrated varying grades/degrees of a wide spectrum of toxicities. These include nausea, fatigue, xerostomia, nephrotoxicity and haematological toxicity, to name a few. In a systematic review and meta-analysis of 17 original studies, low grade toxicities were the most documented [74]. Anaemia related toxicities were the commonest haematological toxicities occurring in 23% of patients (IQR: 7–35%). Leucopaenia and thrombocytopaenia were recorded in a median of 14.2% (8–25%) and 15% (6–24%) of patients, respectively [74]. Up to 10% of patients experience grade 3 or 4 haematological toxicities [96,97]. This limited bone marrow reserve may be because of prior therapies especially considering the current scheduling and application of this radionuclide therapy. Nephrotoxicity was evaluated in 10 studies with 9.5% of patients (IQR: 0–20%) experiencing renal impairment as a result of RLT. Salivary gland toxicities in the form of pain, swelling and dry mouth post therapy were described in as many as 14.5% of patients [74]. Despite all these toxicities, this therapy is effective and safe even in older patients (>75 years) [98]. In the TheraP randomized, open label, phase 2 trial comparing ^177^Lu-PSMA-617 to cabazitaxel, the safety profile of ^177^Lu-PSMA-617 was superior to that of cabazitaxel. Patients randomized to ^177^Lu-PSMA-617 had fewer reported grade 3–4 adverse events except thrombocytopaenia. This further supports the use of ^177^Lu-PSMA-617 in patients who are older or who have co-morbidities [59].

### 7.6. Rechallenge after Initial Therapy with ^177^Lu-PSMA

Almost a third of patient treated with ^177^Lu-PSMA do not respond to this form of therapy, whilst some patients who demonstrated an initial excellent response will relapse. Rechallenge of an oncologic treatment in patients with mCRPC who initially responded to the primary treatment has been described as a potential treatment option after a treatment “holiday”. This concept has been described for the chemotherapeutic drug with reasonable responses and moderate toxicity. Gafita et al. described their initial experience with ^177^Lu-PSMA rechallenge in eight patients who underwent a median of 2 cycles (range 1–4). There was a treatment (^177^Lu-PSMA) free period of 5.4 months (range: 3.8–14.7 months). Almost 40% (3/8) of the patients has a biochemical response with a PSA decrease of 50% [99]. There was an encouraging radiographic response in three patients while 4 patients had progressive disease. The median OS and PSA PFS were 14 (95% CI: 6.2–21.8) and 3.2 (95% CI: 2.6–3.7) months, respectively. While there is evidence of that retreatment with ^177^Lu-PSMA is feasible and efficacious, it does come at a risk of higher toxicities. In their cohort, Gafita et al. reported grade 3 toxicities in 3 patients. A study with a larger number of participants confirmed these findings [100]. The patients were initially treated with a median of three cycles (range: 1–5 cycles) and went on to receive another 3 cycles (range: 1–5 cycles) of retreatment. The median cumulative administered activities at baseline therapy and, after rechallenge were 17.9 GBq and 37.4 GBq, respectively. There were no grade 4 toxicities noted with grade 3 toxicities in 23% of the patients which included: anaemia (3 patients), leucopaenia (2 patients), thrombocytopaenia (1 patient), neutropaenia (1 patient) and one patient developed renal impairment. The results demonstrated that majority of the patients benefitted from additional cycles. There was a stable PSA level (<25% increase and <50% decrease) in 50–60% of the patients. A ≥50% decline in PSA was seen in 26% of patients after the first cycle with 40% and 20% declines in PSA seen after the second and third cycles of rechallenge, respectively. After a median of 3 months (range: 1–15 months), the median OS was 12 months after the retreatment which was lower than the 25 months OS after the primary therapy. The median time PSA progression was 2.8 months (range: 1–11 months). Violet et al. also found that 11/15 (73%) of patients had a PSA decline of atleast 50% after retreatment with ^177^Lu-PSMA at first or second relapse [94]. The median OS from the time of study enrollment was 26.6 months for patients that had retreatment [94]. This is comparable to the results of Yordonova et al. Contrary to the other two studies, there were grade 4 toxicities documented namely thrombocytoapenia and neutropaenia.

The results from these studies prove that rechallenge in patients that had an initial response to ^177^Lu-PSMA is feasible and should be considered in select cases. In view of the toxicities after rechallenge, ideally patients should have recovered from any toxicity resulting from the initial treatment with ^177^Lu-PSMA or conventional systemic therapies. This decision should be made in a multidisciplinary tumour board setting with input from all managing physicians.

## 8. Actinium-225 Therapy

The potential of Actinium-225 (^225^Ac) for the treatment of advanced cancers has been proven in several clinical trials. It is produced from a Thorium-229 (^229^Th) generator, however, there have been attempts of large-scale linear accelerator or cyclotron production by irradiation of Radium-226 (^226^Ra) [101]. It is an alpha emitting particle with a relatively long half-life of 9.9 days which make ^225^Ac a cytotoxic radionuclide. ^225^Ac is classified as a “nanogenerator” or “in-vivo generator” as the decay yields four alpha particles, three β^−^ disintegrations and two gamma emissions [101,102,103]. There is a low abundance of gamma emissions which may be used for dosimetric calculations although their low abundance renders ^225^Ac unsuitable for planar SPECT imaging. While most authors do not do posttherapy scans following administration of ^225^Ac, some authors have reported acquisition of posttherapy scans using the 440 kEV y-coemission of ^213^Bi (26% probability), the 218 keV y-coemission of ^221^Fr (12% probability) and the Brehmstrahlung of ^209^Pb [104]. The alpha particles that are produced have energies ranging from 5.8–8.4 MeV with soft tissue ranges of 47–85 µm. This soft tissue penetration is equivalent to 2–3 cell diameters, which results in selective killing of targeted cells while sparing the surrounding tissues [105]. The high linear energy transfer of alpha particles deposited within a short radius cause irreparable double strand DNA breaks [106]. Beta particles are heavily dependent on the phase in the cell cycle as well as the tissue oxygenation status (production of free radicals) for the induction of DNA damage. Alpha particles on the other hand are larger in size as compared to beta particles which allows for direct DNA damage independent of the oxygenation status.

### 8.1. Efficacy of ^225^Ac-PSMA Therapy for mCRPC

In contrast to ^177^Lu, there are fewer clinical studies and trials on the efficacy of ^225^Ac in the treatment of patients with mCRPC. This may be related to the issues of production costs, logistical and supply problem of alpha emitters. Preclinical and preliminary data on the efficacy and safety of PSMA targeted alpha therapy (TAT) are encouraging [14,107].

The Heidelberg group published their initial experience of ^225^Ac as salvage therapy in two patients who had been heavily pretreated and with one of the patients having progressed following ^177^Lu-PSMA therapy. The patient who had progressed following ^177^Lu-PSMA therapy had liver metastases which has been considered a poor prognosticator in studies using ^177^Lu-PSMA therapy and has since been considered a criterion for exclusion in ^177^Lu-PSMA therapy trials. This patient responded well to ^225^Ac therapy with his serum PSA level dropping from 294 ng/mL to undetectable following three cycles of therapy [108]. In both patients there was an excellent biochemical and imaging response with none or limited bone marrow toxicities. The most significant side effect from this therapy in both patients was xerostomia. This work provided encouraging results and demonstrated the potential of ^225^Ac in patients with mCRPC who have exhausted all other options.

**Figure 3:**
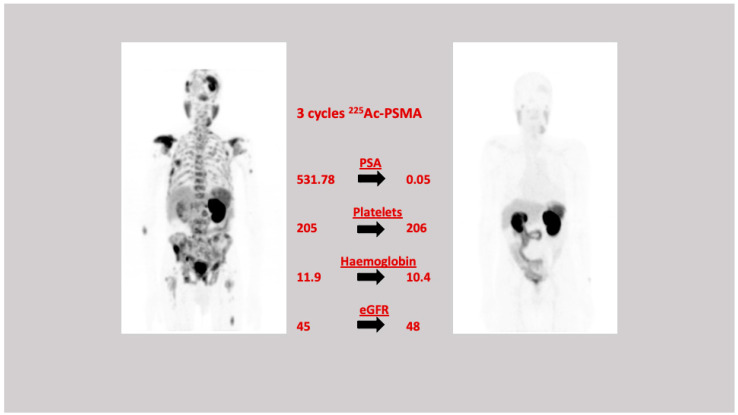
A 79 yr old male with prostate adenocarcinoma. Gleason score = 4 + 3. Previous therapy—prostatectomy. ^68^Ga-PSMA-617 demonstrated widespread axial and appendicular skeletal metastases. He received three cycles of ^225^Ac-PSMA therapy (19 July 2017, 28 September 2017 and 23 November 2017). The patient had an excellent biochemical, clinical, and imaging response and was in remission.

The evidence presented thus far on targeted alpha therapy (TAT) with ^225^Ac-PSMA in mCRPC patients is in heavily pre-treated patients who have exhausted all treatment options. In seventeen chemotherapy naive men with mCRPC, Sathekge et al. demonstrated an impressive response to ^225^Ac-PSMA-617 with 70% of these patients having a ≥80% decline in serum PSA after a single cycle of therapy and 82% of patients having a ≥90% decline in serum PSA level at the end of treatment. Only three patients had a rise in the serum PSA, however one of these patients went on to eventually have a serum PSA decline of 74% by the end of treatment, while the other patient progressed despite having received three cycles of therapy and subsequently received no further treatment. After a median follow-up of 13 months, just over 40% of the patients had remained in remission. While this follow-up period is limited especially for monitoring long-term responses and toxicities, the outcome in these patients is still quite impressive considering that progression free survival with some of the chemotherapeutic drugs is only up to 4.8 months [109]. Figure 3. An image of a chemo-naïve patient with a remarkable response to targeted alpha therapy with ^225^Ac-PSMA. This study presents evidence that supports the early introduction of TAT in the therapeutic schema of mCRPC. There is a need for phase 3 clinical trials to better define the timing of the application of ^225^Ac-PSMA in the treatment sequence of mCRPC. In a larger series of >70 patients, this group went on to confirm these findings of an excellent biochemical and imaging response in heavily patients with mCRPC who had failed other conventional therapies. In this study, 70% of patients had a PSA decline of ≥50% and this was the single most significant predictor of overall survival with an OS of 20.1 months for patients with serum PSA decline of ≥50% after the first treatment cycle and 10.5 months for those with a <50% decline in serum PSA level [110]. They also found that previous treatment with ^177^Lu-PSMA was associated with a shorter progression-free survival suggesting that prior therapy with this beta emitter may induce resistance to radiation [110].

In a detailed assessment of duration of tumour control from any 1st, 2nd, 3rd, and 4th line therapy. In this analysis of 40 men, they found that irrespective of the treatment modality, the median tumour control duration was 8, 7, 6 and 4 months. The median duration to arbiraterone, docetaxel, enzalutamide, carbazitaxel and ^223^Ra irrespective of treatment line was 10, 6.5, 6 and 4 months, respectively [111]. Radioligand therapy with ^225^Ac-PSMA which was applied as last line therapy had a mean tumour control duration of 9 months which is quite remarkable as the general observation was that the duration of tumour control decreased the further down the line the treatment was applied [111]. The findings in this study together with those of Sathekge et al. in their chemotherapy naïve patients, leave an unanswered question as to whether there would be greater benefit if ^225^Ac-PSMA was given earlier in the treatment line. This can only be interrogated in clinical trials. The case report by Rathke et al. of a patient with a 5-year duration of remission, supports this finding of prolonged duration of tumour control with ^225^Ac [112].

A group from the Netherlands looked at the clinical outcomes and molecular profiling of advanced mCRPC in patients treated with ^225^Ac-PSMA-617. They performed immunohistochemical staining and next generation sequencing of the pre- and post-therapy (^225^Ac-PSMA) biopsy specimen. When a metastatic biopsy was unavailable, they utilized archived biopsy specimen. The staining performed was for neuroendocrine markers (CD56 antigen, chromogranin and synaptophysin), PSMA, androgen receptor and Ki-67 expression. In terms of efficacy, their findings confirm those of other published work. PSMA RLT-naïve patients had a longer median OS 12.6 months as opposed to 1.3 months for the patients who previously underwent ^177^Lu-PSMA RLT. Patients with low baseline PSMA expression H-scores (<200; *n* = 2) had worse prognosis than patients with higher H-score (≥200; *n* = 11) with a median OS of 1.8 and 12.6 months, respectively. The H-scores were inversely correlated to the Ki-67 index [113]. The two patients with BRAC1 mutation displayed longer survival compared to those without DNA damage repair alterations. Indeed, while it is not possible and ethically justifiable to collect specimen from every metastatic site, the results from this small study do demonstrate that individual biomarkers need to be considered when planning or selecting therapy. Larger studies are necessary to confirm these findings.

As more centers gain access to ^225^Ac-PSMA, more data is becoming available. The published studies to date further highlight and demonstrate the promising PSA response rates as well as the survival benefit of ^225^Ac-PSMA as salvage therapy for patients with mCRPC who may have exhausted all other treatment options, are ineligible or refuse the standard of care treatment regimen [114,115,116,117]. The presence of visceral metastasis has been associated with a poor prognosis [115], however exceptional responses with long lasting tumour control have been noted in patients with visceral metastasis treated with ^225^Ac-PSMA [112,118,119].

### 8.2. Dosimetry

Internal dosimetry refers to the calculation of the amount and the distribution of radiation absorbed by organs in the body from a particular radionuclide [120]. Patients-specific dosimetry takes into account the individual patients physical characteristics (e.g., body habitus, etc.) and the measured radiopharmaceutical kinetics in that individual, which makes for personalized or precision medicine [121]. The idea is to deliver the maximum dose to the target tumour tissue while sparing or ensuring that the dose to the surrounding tissues is as low as reasonably achievable. The acquisition of post therapy scans from dosimetric calculation is hardly implemented following therapy with ^225^Ac-PSMA. There is resultant low abundance of gamma photons emitted from the daughter products in the decay scheme of ^225^Ac. This is because the radiopharmaceutical activities administered are quite low, in fact a thousand times lower than that administered for ^177^Lu-PSMA therapy. Kratochwil and colleagues estimated organ-absorbed doses for ^225^Ac-PSMA-617 by extrapolating pre-existing ^177^Lu-PSMA-617 data to the half-life of ^225^Ac. The salivary glands, kidneys and red marrow were found to be potential dose-limiting organs. Assuming a biologic effectiveness of 5, dose estimates from 1 MBq ^225^Ac-PSMA-617 were 2.3 Sv, 0.7 Sv and 0.05 Sv for salivary glands, kidneys, and red marrow, respectively [108]. The corresponding radiation absorbed doses for the salivary glands, kidneys, and red marrow for ^177^Lu-PSMA-617 were 1.38 Gy/GBq, 0.75 Gy/GBq, and 0.03 Gy/GBq, respectively [122]. Upon close analysis, it is evident that the salivary glands receive almost twice as much radiation dose with ^225^Ac-PSMA than with ^177^Lu-PSMA. It has been purported that the dose to normal bone marrow when treating skeletal metastases is lower with ^225^Ac-PSMA than with ^177^Lu-PSMA because of the shorter path length that alpha particles traverse.

### 8.3. Toxicity

The literature on therapy of mCRPC with ^225^Ac-PSMA does not report on a pre-defined protocol for dose estimation as most of the studies were not in a clinical trial setting so the physician determined the dose based on individual patient characteristics such as disease extent as determined by pre-therapy imaging and baseline laboratory results. Following their initial report with two patients, Kratochwil and colleagues set out to determine the ideal dose that bridges the compromise between efficacy and toxicity. Administered doses ranged from 50 kBq/kg body weight with escalations of 50 kBq/kg body weight up to a total of 200 kBq/kg body weight. They found that with increasing doses per kg body weight, the toxicity profile also amplified. While higher doses demonstrated great anti-tumour effects, one patient receiving 150 kBq/kg and all patients receiving 200 kBq/kg discontinued therapy or requested a dose reduction as the side effects became unbearable. A dose greater than 100 kBq/kg resulted in xerostomia which became intolerable at doses above 150 kBq/kg per cycle, therefore, 100 kBq/kg body weight was the dose that resulted in effective tumour control with minimal off-target toxicity [108]. This study is not powered by sufficient patient numbers but a few publications on ^225^Ac-PSMA seem to use these results for the justification of the dose determination [114].

There are several ^225^Ac-PSMA related side-effects ranging from dry eyes, anorexia, dyspepsia, nausea and vomiting, constipation, and fatigue, to name a few [108,110,115]. The vast majority of publications report on the major treatment related toxicities (xerostomia, haematological toxicity and renal toxicity) which will be discussed briefly below.

*Xerostomia*: Xerostomia is the most frequently encountered side effect and is by far the commonest reason for discontinuation of ^225^Ac-PSMA [108,110,115]. This is the area of extensive research as the pursuit for ways to reduce salivary gland toxicity continues. A few interventions have been attempted with limited success. These include external cooling with ice packs applied over the salivary glands (parotids and submandibular), injection of Botulin toxin, the co-administration of monosodium glutamate as well as saline irrigation and steroid injections [123,124,125,126]. Considering the tumour sink effect, the de-escalation method first described and implemented by Sathekge and colleagues is one intervention that seems to have success. The patients are treated with an initial dose of 8 MBq of ^225^Ac-PSMA with subsequent doses being titrated against biochemical and imaging (^68^Ga-PSMA PET/CT) findings. A follow-up dose of between 4–6 MBq is administered if there has been an acceptable response to the first treatment cycle. The South African group of patients had grade 1 and 2 toxicities while none of the patients discontinued therapy because of xerostomia [110]. Another method explored for reduction of salivary gland toxicity from TAT is a tandem approach in which low activity ^225^Ac-PSMA (median: 5.3 MBq; range: 1.5–7.9 MBq) is administered in the same week, typically consecutive days as a higher dose of ^177^Lu-PSMA (median: 6.9 GBq; range: 5–11 GBq). No grade 3 or 4 xerostomia toxicities were reported while 40% of the patients developed grade 1 and 25%, grade 2 xerostomia [127]. Of the patients with grade 2 xerostomia, two patients had already developed grade 1 xerostomia from previous ^177^Lu-PSMA therapy. The results from this tandem approach are similar to those from the de-escalation method. Another group found an even lower (13%) rate of grade 1/2 xerostomia using the tandem approach [128]. This tandem approach results in satisfactory tumour control with reduced toxicities and should be explored in larger studies or trials. While adjustments of the technical/practical aspects of therapy administration have shown reduced toxicities, other researchers have looked at improving the molecular aspects of the PSMA ligands in order to reduce off-target binding. One such amendment has been described by Benesova et al., in which an albumin-binding moiety altered the biodistribution of PSMA radioligands [129,130]. In pre-clinical work, this modification resulted in elevated accumulation in the tumour with fast clearance from the background organs, therefore reducing residency time, which would result in reduced toxicities [129,130].

*Haematological toxicity*: The skeleton is the commonest site of distant metastases in PCa, especially in the setting of mCRPC. The physical properties of ^225^Ac-PSMA such as high liner energy over a short path make it a better agent for therapy of diffuse bone marrow metastases. All patients had skeletal metastases in the study by Feuerecker et al. They reported a lower frequency of grade 4 haematological toxicties. The grade 3 haematological toxicities were as follows: 8 patients had anaemia (31%, 95% CI: 16–50%), three had thrombocytopaenia (12%, 95% CI: 3–29%) and leucopaenia in 7 patients (27%, 95% CI: 13–46%), while Grade 4 toxicities were anaemia in one patient (4%, 95% CI: 0–20%), thrombocytopaenia in two patients (8%, 95% CI: 1–25%) and none of the patients had grade 4 leucopaenia [115]. In the largest cohort to date, Sathekge et al. reported no grade 4 haematological toxicities and few grades 3 anaemia, thrombocytopaenia and leucopaenia toxicities in 7%, 1% and 3% of their patients [110]. The differences in the two study groups may be because the patients in the work by Feuerecker et al. had been heavily pre-treated and all patients had received prior ^177^Lu-PSMA which may have already compromised the bone marrow function. This is something to be borne in mind in the cases of patients that are at the end of the treatment line and have exhausted all options as is the case in most patients referred for ^225^Ac-PSMA.

*Renal toxicity*: The PSMA protein is found in the tubular cells of the kidney. A case report of two patients described the long-term renal toxicity following treatment with ^225^Ac-PSMA [131]. While the two patients in this report had prior or underlying renal injury, the authors attempted to provide convincing evidence implicating ^225^Ac-PSMA therapy by highlighting the temporal relationship between therapy and onset of renal impairment as well as histopathological evidence [131]. This provides supporting data for the careful examination of baseline renal function as patients with chronic kidney disease may develop renal toxicity. Therapy induced nephrotoxicity has also been reported in some studies on ^225^Ac-PSMA therapy with Grade 1/2 renal toxicities observed in 14–25% of patients receiving ^225^Ac-PSMA therapy [110,114]. The overall impression is that patients with underlying renal impairment had higher grade nephrotoxicity. Long term follow-up of renal function is advised to monitor for delayed renal toxicity as kidneys are late reacting organs.

*Liver toxicity*: Compared to the other major end organ damage/toxicity, there has been no study thus far demonstrating significant Grade II/IV hepatotoxicity following targeted alpha therapy with ^225^Ac-PSMA in patients with mCRPC [114].

#### Other PSMA Targeted Radionuclude Therapy (RNT) in Development

There is continuous research proceeding in the pursuit of improved targets for mCRPC. These include adjustments made to the antibodies or the use of other alpha radionuclides. Most of these agents are still in the pre-clinical phase of investigation, however, ^213^Bi-PSMA is one of the few to have been tested clinically. In the only documented clinical case thus far, Sathekge et al. treated a patient with mCRPC with ^213^Bi-PSMA and reported on their experience. The patient had progressed on other conventional therapies. Patient received 2 cycles of ^213^Bi-PSMA-617 with a cumulative dose of 592 MBq [132]. There was an overall biochemical, clinical, and imaging response with serum PSA decreasing from 237 μg/L to 43 μg/L. This case highlights the potential of this tracer for targeted alpha therapy in mCRPC. The short half-life means that the residency time is reduced, therefore potentially reducing the likelihood of severe toxicities. In the treatment of neuroendocrine tumours, this radioligand resulted in high numbers of long-lasting anti-tumour responses. In view of the short half-life, a combinatorial approach may be considered with ^213^Bi-PSMA being paired with another radioligand to enhance the activity of this radioligand. Table 1. below gives a list of alpha emitting radionuclides being investigated or in use for targeted alpha therapy.

## 9. Combination Therapies

Drug resistance is one of the main obstacles in the treatment of metastatic PCa. Early metastatic PCa is usually hormone sensitive and responds to ADT, however over the years the addition of docetaxel, abiraterone, apalutamide and enzalutamide has shown improved survival advantage. This was highlighted in the preliminary results of the PEACE 1 phase 3 trial in which patients were randomized to receive either standard of care (SOC—ADT/bilateral orchidectomy and docetaxel), SOC and abiraterone, SOC and radiation therapy or SOC, abiraterone and radiation therapy. In this study it was proven that the addition of abiraterone to the SOC conferred a benefit of delayed radiological progression and progression to castration resistance [140]. This is an example of improved efficacy of combination therapies. Despite great initial responses, resistance to these agents develops quickly. Resistance is a major drawback with most drugs in the treatment scheme of mCRPC and it usually sets in even with second generation androgen axis targeting drugs. Androgen receptor mutations, amplification, splice variants and bypass pathways as well as androgen independent alternative pathways have been identified as mechanisms involved in resistance in PCa [141]. Any one of these mechanisms exclusively or in concert are involved in this drug resistance. This is the reason and justification for combination therapies which may achieve superior tumour control by targeting multiple pathways involved in drug resistance. There have been many clinical studies assessing the combinations of the more conventional life prolonging PCa drugs (CHAARTED and STAMPEDE trials) which demonstrated that therapy combinations prolong survival of patients with metastatic PCa.

Researchers from Australia paired the tumour-specific radiation sensitizer idronoxil (NOX66) with ^177^Lu-PSMA-617 (LuPIN) with the hope to improve outcomes in heavily treated mCRPC. In this Phase I/II study the researchers divided the patients into two groups receiving different doses of NOX66. The first cohort of eight (8) patients received 400 mg NOX66 suppositories on days 1–10 of a 6-week cycle while the second cohort of twenty-four (24) patients received 800 mg NOX66 suppositories on the same schedule [142]. After a median of five cycles, there was a biochemical (PSA) response in almost two-thirds (63%) of the patients, with 91% of the patients having any decline in PSA. The median PSA PFS was 6.1 months (95% CI: 2.8–9.2) with a median overall survival of 17.1 months (95% CI: 6.5–27.1). The most frequent adverse effects (non-specific grading) were anaemia (88%), xerostomia (59%) and fatigue (69%). Nine (28%) patients also reported grade 1 and 2 anal inflammation as a result of the NOX66 suppositories [142]. In this dose escalation study, it is evident that the combination therapy was well tolerated with no increase in toxicity of ^177^Lu-PSMA-617. While this is encouraging, these results are similar to those found in single agent ^177^Lu-PSMA trials/studies without the additional adverse effects related to the suppositories. At this point there appears to be no added advantage from the combination therapy.

In addition to upregulating PSMA messenger RNA production and PSMA receptor density on the cell surface, but androgen receptor blockade may also be a radiosensitizer. A phase 2 multicenter trial testing enzalutamide and ^177^Lu-PSMA-617 vs. enzalutamide monotherapy (ENZA-P, ANZUP 1901; NCT04419402) is in the recruitment phase and aims to recruit 160 patients with a 1:1 randomization. Patients randomized to the combination treatment arm will receive 4 cycles of 7.5 GBq ^177^Lu-PSMA-617 every 6 weeks in together with 160 mg enzalutamide daily. The primary outcome measure will be PSA-PFS with radiographic PFS (rPFS), PSA response rate, pain response, clinical PFS (cPFS), health-related quality of life and lastly frequency and severity of adverse events.

Another ongoing trial is the ^177^Lu-PSMA-617 Therapy and Olaparib in patients with mCRPC (LuPARP; NCT03874884). Poly Adenosine diphosphate Ribose Polymerase (PARP) inhibitors play an important role as radiosensitizers as they help minimize and block the catalytic and DNA reparative role of the PARP enzyme. This is a single arm study where patients will receive up to four cycles of 7.4 GBq ^177^Lu-PSMA-617 and Olaparib (PARP inhibitor) on days 2–15 of each cycle. This study also will also have a dose escalation component where varying doses (range: 50–300 mg twice daily) of Olaparib are administered. The primary outcome is to calculate the maximum tolerable doses and dose limiting toxicities. It is envisioned that the synergistic activity of these two therapeutic regimens will result in improved and sustained patient outcomes because evidence has been emerging suggesting that there is an interaction between the immune system and the tumour DNA damage. This therapy would be more beneficial in patients with proven BRCA1/2 mutations.

Another exciting prospect is combining ^177^Lu-PSMA with immune checkpoint inhibitors. Immune checkpoint inhibitors such as anti-programmed death 1 (anti-PD-1) monoclonal antibodies (nivolumab and pembrolizumab) and anti-CTLA-4/CD28T monoclonal antibodies enhance T cell effector function and have found application in many malignancies including melanoma and lung cancers [143,144]. Immunotherapeutic drugs that can be combined with ^177^Lu-PSMA or ^225^Ac-PSMA include anti-PD1/antiPD-L1 and anti-CTLA-4 + anti-PD-1 to name a few [145]. Early preclinical work assessing the synergistic activity of ^225^Ac and PD-1 demonstrated an improved therapeutic efficacy (OS: 51.5 days) as opposed to when either was used as monotherapy (anti-PD-1: 37 days and RNT: 32 days). The time to progression was prolonged for the combination group (47.5 days) than with the anti-PD-1 (33.5 days) and RNT group (30 days) [145]. The combination of anti-PD-1 and RNT synergistically reduces tumour burden and improves the time to progression and overall survival. This pre-clinical work offers promising results and to this end, the phase 1b/II PRINCE trial (NCT03658447) has been registered. It aims to test efficacy, safety, and tolerability of the combination of pembrolizumab with ^177^Lu-PSMA-617 in patients with mCRPC. Patients will receive 200 mg pembrolizumab given 3-weekly for up to 35 cycles and 6-weekly ^177^Lu-PSMA-617 therapy up to 6 cycles starting at 8.5 GBq and reducing the dose by 0.5 GBq at every subsequent cycle. This therapy may only be feasible in a select few with a high load of neoantigens, therefore further interrogation of the tumour microenvironment may offer insight to the application of these therapies in PCa.

The trials reported thus far aim to combine a novel agent/drug with radionuclide therapy to enhance the therapeutic effect. Other trials include combination of PSMA labelled radionuclide therapies with immune checkpoint inhibitors and some have even attempted combinations of radionuclide therapies such as ^177^Lu-PSMA small molecule with radiolabelled antibody J591. These combinations are trying to improve efficacy while reducing off target toxicities. Other classes of novel treatments such as androgen receptor (AR) degraders are still in clinical development. Further interrogation of these novel treatment agents, either alone or in combination with prostate-cancer targeting therapies, will be essential to frame optimal management strategies for this challenging disease. The landscape of treatment options thus continues to expand for patients with mCRPC.

## 10. Future Perspectives

It is evident that radionuclide therapy with alpha or beta particles has an impact on survival in men with mCRPC, however There is a need to look at improved methods of increasing the tumor target binding whilst reducing the normal tissue (off-target) binding. To this end several researchers have looked at developing antibody or albumin based PSMA targeting agents both of which alter the biodistribution of these tracers with possibilities of reducing xerostomia and dry eye effects [129,130]. A phase 1 trial testing ^225^Ac-J591 (which is an antibody-based imaging) has been initiated. The study recruited 22 patients and varying doses were administered with a determined safe dose of 93.8 kBq/kg. Over two thirds (14/22) of the patients experienced any PSA decline with 9 (41%) of the patients having a PSA decline of >50% [146]. The multicenter expansion trial stemming from this study is already underway. Small molecules with an albumin binding moiety are thought to improve tumour retention of PSMA radioligands whilst reducing residence time in normal tissues. This may translate to dose reduction and adjusted frequency of therapy. The pre-clinical work has demonstrated improved pharmacokinetics and efficacy when labelled to ^177^Lu or ^225^Ac [129,130]. The first clinical studies with these modified PSMA ligands have demonstrated great promise but further modifications and trials are still necessary to optimize tumour retention while reducing non-tumour uptake which leads to significant morbidity [147,148]. The optimization strategies and the clinical trials on combinatorial therapies are welcome development and the outcomes of these are eagerly anticipated.

## 11. Conclusions

Despite developments in screening and diagnosis, PCa remains a significant cause of morbidity and mortality in men. There is increasing evidence supporting the use of molecular targets for imaging and therapy. Accumulating data is showing the improved accuracy of PSMA labelled tracers for imaging PCa. This has catapulted the acceptance of these tracers for guiding therapy. The availability of PRLT as a treatment option for the challenging pathology of mCRPC has encouraged researchers to better define the role and timing of this therapy. The results thus far have demonstrated good efficacy when used as salvage therapy. ^177^Lu-PSMA has shown great PSA responses and with the results of the phase three VISION trial finally out, it is anticipated that ^177^Lu-PSMA will get approval for treatment in selected patients with mCRPC. ^225^Ac-PSMA has also resulted in great responses when used as salvage therapy. The physical characteristics of ^225^Ac-PSMA result in more remarkable PSA responses, nevertheless at the risk of severe salivary gland toxicity. Supply challenges of ^225^Ac-PSMA as well as significant xerostomia are issues that need to be addressed for wider clinical acceptance. Ligand modifications and combinatorial therapies are promising areas of continued research. With all these developments happening in the field of PCa, we can only look forward to what promises to be an exciting era in PCa theranostics.

## Figures and Tables

**Table 1:** An overview of alpha emitting radionuclides under both investigation clinical use for targeted alpha therapy for mCRPC [132,133,134,135,136].

Radionuclide	Half-Life	Production Method	Energy	Range in Tissue	LET (keV/um)	Ability to Image	Animal Model/No. of Patients	Small Molecule or Antibody	Outcome/Results	Reference
Preclinical										
^211^At (^211^At-VK-02-09-Lu)	7.2 h	Cyclotron	6.9 MeV	55–80	71–230	No	Mice: PC3-ML-Luc	Small molecule	A dose-dependent therapeutic effect in flank xenograft and metastatic tumor models of prostate cancer. High stability in vivo, and rapid clearance from off-target tissues in mice including kidneys, salivary and lacrimal glands	[133]
^212^Pb/^212^Bi (^212^Pb-NG001 + ^212^Pb-PSMA-617)	10.6 h	Cyclotron/generator	7.9 MeV	40–100	61–230	Yes	Mice: C4-2	Small molecule	Good tumour uptake with reduced off-target binding Genitourinary excretion No difference between the biodistribution of ^212^Pb and ^212^Bi during the 24-h period No report on efficacy	[134]
^149^Tb (^149^Tb-PSMA-617)	4.12 h	Accelerator	3.97 MeV	25	140	Yes	Mice: PC-3-PIP PC-3 flu	Small molecule	Mice with two doses showed better tumour growth inhibition Higher quantities of activity ± more frequent injections may be necessary for tumour eradication May be used for imaging as well “alpha PET”	[135]
^227^Th (PSMA-TTC, BAY 2315497)	18.7 days	Generator	32.8 MeV	50–70	71–230	Yes	Mice MDA-Pca-2b cell, LNCaP, 22Rv1, C4-2 and patient derived xenograft	Antibody	Antitumour activity in hormone sensitive and hormone-resistant tumours was dose dependent Selective uptake in tumour for >21 days	[136]
Clinical										
^225^Ac (^225^Ac-PSMA-617)	9.9 days	Cyclotron/accelerator	27.9 MeV	47–85	61–230	Yes	2 patients	Small molecule	PSA decline to undetectable levels. Complete response on imaging.	[104]
^225^Ac (^225^Ac-PSMA-617)	9.9 days	Cyclotron/accelerator	27.9 MeV	47–85	61–230	Yes	14 patients	Small molecule	Dosimetry study. Mean doses assuming RBE_5_: Salivary glands = 2.3 Sv, Kidneys = 0.7 Sv, and Red marrow = 0.05 Sv Xerostomia dose limiting toxicity at doses >100 kBq/kg per cycle	[108]
^225^Ac (^225^Ac-PSMA-617)	9.9 days	Cyclotron/accelerator	27.9 MeV	47–85	61–230	Yes	7 patients	Small molecule	Genetic analysis. Patients with resistance to PSMA-TAT despite positivity, harbor mutations in DNA damage repair and checkpoint genes.	[137]
^225^Ac (^225^Ac-PSMA-617)	9.9 days	Cyclotron/accelerator	27.9 MeV	47–85	61–230	Yes	40 (38) patients	Small molecule	Duration of tumour control. Median duration of tumour control of ^225^Ac-PSMA-617 as last line therapy was 9 months. Median duration of tumour control with abiraterone (1st line) = 10 months, docetaxel (2nd line) = 6.5 months, enzalutamide (3rd line) = 6.5 months and carbazitaxel (4th line) = 6 months	[111]
^213^Bi (^213^Bi -PSMA-617)	45.6 min	Generator	8.5 MeV	40–100	65–230	Yes	1 patient	Small molecule	PSA decline ≥ 50% Clinical and imaging response (11 months post therapy)	[132]
^225^Ac (^225^Ac-PSMA-617)	9.9 days	Cyclotron/accelerator	27.9 MeV	47–85	61–230	Yes	17 patients	Small molecule	Chemotherapy naïve patients. Reduced toxicity due to de-escalation method	[109]
^225^Ac (^225^Ac-PSMA-617)	9.9 days	Cyclotron/accelerator	27.9 MeV	47–85	61–230	Yes	73 patients	Small molecule	Predictors of disease-free survival and overall survival in mCRPC. Multivariate analysis: prior ^177^Lu-PSMA therapy and PSA decline ≥ 50% associated with PFS	[110]
^225^Ac (^225^Ac-PSMA-617)	9.9 days	Cyclotron/accelerator	27.9 MeV	47–85	61–230	Yes	38 patients	Small molecule	No difference in response rates and survival between patients with visceral metastases vs. those with bone and lymph node metastases	[138]
^225^Ac (^225^Ac-PSMA-617)	9.9 days	Cyclotron/accelerator	27.9 MeV	47–85	61–230	Yes	28 patients	Small molecule	PSA progression poor prognostic factor of OS. Any PSA decline good prognostic factor of PFS.	[114]
^225^Ac (^225^Ac-PSMA-617)	9.9 days	Cyclotron/accelerator	27.9 MeV	47–85	61–230	Yes	26 patients	Small molecule	Liver metastases associated with shorter PSA PFS	[115]
^225^Ac (^225^Ac-PSMA-I&T)	9.9 days	Cyclotron/accelerator	27.9 MeV	47–85	61–230	Yes	14 patients	Small molecule	^225^Ac-PSMA-I&T showed promising antitumour effect which is comparable to ^225^Ac-PSMA-617.	[139]
^225^Ac (^225^Ac-PSMA-617)	9.9 days	Cyclotron/accelerator	27.9 MeV	47–85	61–230	Yes	13 patients	Small molecule	Clinical efficacy and molecular profiling. Patients with low baseline PSMA expression H-scores (<200, *n* = 2) had worse OS compared to patients with H-scores ≥200, *n* = 11 (median OS = 1.8 months vs. 12.6 months) Longer survival in patients with BRAC1 gene DNA damage repair alterations (16.1 vs. 7.6 months)	[113]
